# Molecular profiling of coronary stent restenosis: A systematic review and functional analysis of implicated genes

**DOI:** 10.1097/MD.0000000000049455

**Published:** 2026-06-26

**Authors:** Rajaa EL Mansouri, Rachida Habbal, Hind Dehbi

**Affiliations:** a Laboratory of Cellular and Molecular Pathology, Medical School, University Hassan II, Casablanca, Morocco; b Department of Cardiology, University Hospital Center Ibn Rochd, Casablanca, Morocco.

**Keywords:** bioinformatics, coronary stent restenosis, drug-eluting stents (DES), enrichment analysis, extracellular matrix re modeling, gene expression, inflammation

## Abstract

**Background:**

Coronary stent restenosis occurs in approximately 5% of patients treated with drug-eluting stents (DES) and is associated with adverse clinical outcomes. Elucidating the genetic mechanisms underlying restenosis may support precision medicine approaches to improve patient management.

This systematic review aimed to synthesize evidence on genes and biological pathways associated with DES-related restenosis and to perform functional analysis of the implicated genes using bioinformatics tools.

**Methods:**

The review was conducted according to Preferred Reporting Items for Systematic Reviews and Meta-Analyses Extension for Scoping Reviews guidelines. A systematic search of PubMed, Scopus, and Web of Science was performed for human studies investigating genetic or genomic factors in coronary restenosis, with the last search conducted in March 2024. Eligibility criteria included original studies reporting genetic associations with DES restenosis. Screening and data extraction were performed by a single reviewer. Identified genes underwent gene set enrichment analysis using *Enrichr* (Ma’ayan Laboratory, Computational Systems Biology) and *ClueGo* extension on *Cytoscape* (National Resource for Network Biology).

**Results:**

Seventeen studies met the inclusion criteria. The studies highlighted multiple genes involved in extracellular matrix remodeling, inflammatory signaling, and the renin-angiotensin system. Gene enrichment analysis confirmed the overrepresentation of these biological pathways in DES-associated restenosis.

**Conclusions:**

This systematic review synthesizes the genetic and molecular contributors to DES-associated restenosis and identifies potential targets for future research and personalized therapies. No external funding was received, and the protocol was not registered.

## 1. Introduction

Percutaneous coronary intervention (PCI) has profoundly transformed the management of coronary artery disease, shifting myocardial revascularization from a predominantly surgical discipline to a catheter-based approach.^[1]^ Since the introduction of coronary balloon angioplasty by Andreas Grüntzig in the late 1970s, successive technological innovations have sought to overcome the biological and mechanical limitations of coronary dilation.^[2–4]^ Early bare-metal stents (BMSs) represent major advances in preventing acute vessel closure and elastic recoil following balloon angioplasty.^[5]^ However, the long-term efficacy of BMSs is substantially limited by restenosis, which affects approximately 20% to 40% of patients and frequently necessitates repeat revascularization procedures.^[6]^

The development of drug-eluting stents (DESs), which combine a metallic scaffold with a polymer-based drug delivery system to locally release antiproliferative agents,^[7]^ marked an advancement in the field because they significantly reduce late lumen loss and in-stent restenosis (ISR).^[8]^ As a result, DESs have rapidly become the default strategy for most PCI procedures.^[9]^ Nevertheless, 1st-generation DESs, while demonstrating comparable rates of mortality and myocardial infarction to those of BMSs across a wide range of clinical and lesion subsets, have raised concerns regarding late and very late stent thrombosis.^[10,11]^ These safety signals have prompted prolonged dual-antiplatelet therapy and accelerated efforts to refine stent design and polymer biocompatibility.^[12]^ Second-generation DESs, characterized by thinner struts and more biocompatible or bioabsorbable polymers, have since shown superior safety and efficacy compared with both 1st-generation DESs and BMSs.^[13,14]^ The improved performance of 2nd-generation DESs has expanded the scope of PCI into increasingly complex clinical settings, including acute myocardial infarction,^[15]^ diabetes mellitus,^[16]^ chronic total occlusions,^[17]^ bifurcation lesions,^[18]^ and saphenous vein graft disease,^[19]^ as well as selected cases of left main or multivessel coronary disease.^[20]^ In parallel, evolving evidence suggests that shorter durations of dual-antiplatelet therapy may be sufficient after implantation of contemporary DESs in low-risk patients, whereas extended treatment remains beneficial in those at high ischemic risk,^[21]^ underscoring a paradigm shift towards more individualized post-PCI management.^[22]^

Despite these advances, ISR has not been eradicated and continues to represent a clinically relevant complication in the DES era.^[23]^ Moreover, emerging technologies, such as drug-coated balloons and fully bioresorbable scaffolds, have been developed to address late adverse events, including neo-atherosclerosis, strut fracture,^[24]^ and long-term vascular dysfunction.^[25]^ Restenosis is not merely a failure of device engineering but rather a complex, multifactorial process driven by interactions among procedural factors, clinical characteristics, and host-specific biological susceptibility.^[26,27]^ At the molecular level, the ISR reflects a tightly regulated response to arterial injury involving endothelial dysfunction, inflammatory signaling, vascular smooth muscle cell (VSMC) proliferation and migration, and extracellular matrix (ECM) remodeling.^[28]^ These processes are orchestrated by interconnected genetic pathways, suggesting that interindividual variability in restenosis risk may be partially genetically determined.^[29]^ Over the past 2 decades, numerous candidate genes and association studies have explored the relationships between single-nucleotide polymorphisms (SNPs) and ISR,^[26]^ identifying genes involved in inflammation,^[30]^ cell cycle regulation,^[31]^ thrombosis,^[32–34]^ and other processes. However, the interpretation of these findings remains challenging because of heterogeneity in study design, sample size, population ancestry, stent platforms, and outcome definitions, resulting in fragmented and sometimes conflicting evidence.

In this context, integrative bioinformatics approaches offer a valuable framework for synthesizing heterogeneous genetic data and enhancing biological interpretability.^[35,36]^ Pathway-oriented analyses and evidence-weighted gene prioritization strategies enable a shift from isolated genetic associations towards a system-level understanding of restenosis pathophysiology. Accordingly, the objective of the present study was to systematically synthesize human genetic evidence related to coronary ISR in the era of DES and to integrate these findings using a biologically informed, evidence-weighted bioinformatics approach. By mapping genetic associations onto established restenosis-related pathways, this work aimed to identify robust molecular determinants of ISR, support risk stratification, and contribute to the development of personalized interventional strategies.

## 2. Methods

### 2.1. Search strategy

The research question guiding this review was developed using the PICO framework, focusing on the genetic etiology underlying ISR among patients treated with DES. Specifically, the review aimed to clarify the association between genetic polymorphisms, particularly SNPs, and the risk of ISR following PCI. The population of interest comprised individuals who had undergone DES implantation for coronary artery disease. The intervention assessed was the presence of specific genetic polymorphisms, with outcomes measured as the incidence of ISR, comparing patients with and without these genetic variants.^[37]^

This study employed a hybrid approach combining elements of systematic review and functional bioinformatics analysis. We conducted a systematic literature search following Preferred Reporting Items for Systematic Reviews and Meta-Analyses (PRISMA) guidelines to identify relevant genetic studies on coronary stent restenosis, while also performing secondary bioinformatics analyses on publicly available gene expression datasets to explore functional pathways and gene enrichment. Because this work integrates narrative synthesis of genetic association studies with computational functional annotation rather than evaluating prospective clinical interventions, the study was not prospectively registered in PROSPERO. This hybrid design allowed us to bridge genetic evidence from the published literature with pathway-level insights derived from bioinformatic tools.

### 2.2. Eligibility criteria

Eligibility criteria were established to ensure the inclusion of relevant and high-quality evidence. Studies were eligible if they were case-control studies, case series, or population-based studies involving patients who received DES, were published up to March 2024, and appeared as peer-reviewed articles or manuscripts. Exclusion criteria encompassed studies focused on RNA variants, incomplete articles, reports without full-text access, and conference proceedings

### 2.3. Outcome measurement

The primary outcomes measured in the included studies were the associations between specific genetic variants and the risk of ISR. Data extracted from each study included the gene name, the SNP investigated, the experimental method used, the type of stent (DES or BMS), the genetic model considered (dominant, recessive, or heterozygous), and statistical findings such as *P* values, odds ratios (ORs), and 95% confidence intervals (CIs). These outcomes allowed for a comprehensive evaluation of the genetic factors potentially contributing to ISR.

### 2.4. Data sources and search strategy

A systematic literature search was conducted using PubMed and Scopus databases, covering publications from 2015 to March 2024. The search strategy incorporated Boolean operators and truncation symbols to maximize sensitivity, using keywords such as “genetic polymorphism,” “SNP,” “target vessel revascularization,” “coronary restenosis,” “ISR,” “DES,” “stent,” “PCI,” “polymorphism, genetic,” “postoperative complications,” “retrospective studies,” and “stroke.” This approach ensured a comprehensive identification of studies relevant to the genetic determinants of ISR.

Study selection was performed independently by 2 reviewers, who 1st screened titles and abstracts for eligibility and then assessed full texts of potentially relevant articles. Any disagreements were resolved by discussion or, if necessary, by consulting a 3rd reviewer. Inter-reviewer agreement was evaluated using Cohen kappa coefficient where available. The process of study selection, including the number of studies excluded at each stage, was documented using a PRISMA flow diagram.^[38]^

### 2.5. Data extraction

Data extraction was carried out using a standardized table to note the key variables from every study. The table included the gene name, the specific SNP being studied, the experimental method used, the type of stent used (DES or BMS), the genetic model considered (dominant, recessive, or heterozygous), and the corresponding statistical findings (*P*-value, OR, and 95% CI). The findings were collated and reported descriptively to define the scope and character of available evidence, and the overall findings were presented in tabular form.

## 3. Quality assessment

Quality assessment and data extraction were conducted to ensure methodological rigor and consistency. Two reviewers independently extracted data using a predefined and piloted extraction table. To further ensure reliability, a subset of articles (10%) underwent double extraction, with discrepancies resolved by consensus. Data synthesis was performed using a framework analysis, generating themes that reflected both the research questions, and the evidence identified in the literature. The entire review process adhered to PRISMA-ScR guidelines and was visually summarized in a PRISMA flow diagram.

Additional quality assessments of the included studies were used by the domain level judgment and *q* genie domain. (see Table S3, Supplemental Digital Content 6).

### 3.1. Ethical review

This study was conducted in accordance with the Declaration of Helsinki. Ethical approval was obtained from the Comité d’Éthique pour la Recherche Biomédicale de Casablanca under reference 18/2023 SRSI/114. The study was also approved by the Ethics Committee of Ibn Rochd University Hospital (or CHU Ibn Rochd). Written informed consent was obtained from all participants (or from their legal guardians when applicable) prior to inclusion in the study.

### 3.2. Bio informatic analysis

#### 3.2.1. Data acquisition and differential expression analysis

Gene expression datasets relevant to IRS were retrieved from the GEO database (https://www.ncbi.nlm.nih.gov/geo/). Differential expression analysis was performed using analytical tool of Gene Expression Omnibus database (https://www.ncbi.nlm.nih.gov/geo/geo2r]), enabling the comparison of gene expression profiles between stented arteries with and without restenosis. Genes demonstrating statistically significant differential expression (adjusted *P*-value <.05) and substantial log2 fold change were selected as candidates for further analysis. Gene symbols were standardized and redundant entries removed prior to downstream functional annotation and pathway analysis.

#### 3.2.2. Gene standardization and redundancy management

To ensure data integrity and minimize redundancy, all gene symbols were standardized according to the HUGO Gene Nomenclature Committee guidelines. Duplicate entries and alternative gene names were consolidated, and only unique gene identifiers were retained for downstream analyses.

### 3.3. Protein–protein interaction network construction

Protein–protein interaction (PPI) networks were constructed using the Search Tool for the Retrieval of Interacting Genes/Proteins database, applying a medium confidence threshold (≥0.3). The resulting interaction networks were visualized in Cytoscape. Densely connected modules within the PPI network were identified using the Molecular Complex Detection (MCODE) algorithm (node score cutoff: 0.2; degree cutoff: 2; max depth: 100).

### 3.4. Functional annotation and pathway enrichment

To systematically interpret the biological significance of differentially expressed genes (DEGs), functional annotation and pathway enrichment analyses were performed using several established bioinformatics resources: gene ontology (GO): GO provides a comprehensive, controlled vocabulary to describe gene product attributes across species, organized into 3 main categories: biological process, molecular function, and cellular component. This framework ensures consistent and reproducible annotation of gene functions, facilitating cross-study comparisons and integrative analyses. Kyoto Encyclopedia of Genes and Genomes (KEGG): KEGG offers curated pathway maps linking gene functions to metabolic and signaling networks, enabling the identification of key biological processes and pathways associated with the gene set under investigation.

### 3.5. Enrichment analyses were conducted using the following platforms

Database for Annotation, Visualization and Integrated Discovery (DAVID) was used to identify statistically overrepresented GO terms and KEGG pathways among the DEGs, providing insights into the functional categories and biological processes most affected. ShinyGO: This user-friendly tool offers an interactive graphical interface for enrichment analysis and supports extensive gene sets, including transcription factor and microRNA targets. ShinyGO at https://bioinformatics.sdstate.edu/go74/ DAVID by providing additional annotation in resources and enhanced visualization capabilities, particularly for human, mouse, and *Arabidopsis* datasets.

GO offers a standardized vocabulary for describing gene functions across species, which facilitates consistency in biological research. Similarly, the KEGG provide online resources that connect gene functions, enzymatic pathways, and genomic data to broaden the understanding of biological processes. To analyze the functions and pathways of genes showing differential expression, researchers can use the DAVID (david.ncifcrrf.gov) to pinpoint relevant GO categories and KEGG pathways. *ShinyGO* is a user-friendly tool that offers a graphical interface for enrichment analysis. While DAVID may encompass a wider range of species, ShinyGO provides more extensive gene sets for transcription factors and microRNA target genes in humans, mice, and *Arabidopsis*. It can be accessed. To investigate the interactions between DEGs, PPI networks can be constructed using the Search Tool for the Retrieval of Interacting Genes/Proteins online database (https://string-db.org/). These networks are built using a medium confidence threshold of ≥0.3. For further network analysis, the MCODE algorithm (version 1.5.1), a Cytoscape plug-in, can be employed to identify densely connected regions based on their topological characteristics. Cytoscape software enables the visualization of the PPI network. MCODE then identifies significant modules within the network and calculates a score for each module. The MCODE analysis typically uses parameters such as a node score cutoff of 0.2, a degree cutoff of 2, a max depth of 100, and the MCODE score.

### 3.6. Gene set enrichment analysis

The Gene Set Enrichment Tool (Enrichr) website and Cytoscape with the ClueGO plug-in were used to generate visual representations of the data. Cytoscape is a software platform used for visualizing complex networks, including protein-protein interaction networks. The ClueGO plug-in, specifically, helps in the functional analysis of gene sets within these networks, allowing for the identification of enriched GO terms and pathways. Enrichr, on the other hand, is a web-based tool that provides a comprehensive set of gene set enrichment analyses, offering insights into the biological functions, pathways, and regulatory mechanisms associated with a list of genes https://maayanlab.cloud/Enrichr/.^[39,40]^ metascape.org^[41]^

### 3.7. Statistical analysis of gene expression and enrichment

Differentially expressed genes were identified based on their log2 fold change (Log FC) and *P* values. The Log FC was used to assess the magnitude and direction of gene expression changes between conditions, with positive values indicating upregulation and negative values indicating downregulation. Genes with higher absolute Log FC values were considered to contribute more significantly to the biological differences observed.

The *P* value was used to determine the statistical significance of the observed expression changes and enrichment results. For enrichment analysis, *P* values were calculated to assess whether specific pathways, ontologies, or biological categories were overrepresented in the input gene list compared to a reference background. A threshold of *P* < .05 was considered statistically significant.

Gene enrichment analysis was performed using the Enrichr platform, and results were visualized through the Apyter interface, which allowed for interactive exploration of enriched terms and categories.

## 4. Results

### 4.1. Systematic review research: genetic determinants of coronary restenosis

#### 4.1.1. Article selection

**Figure 1.**
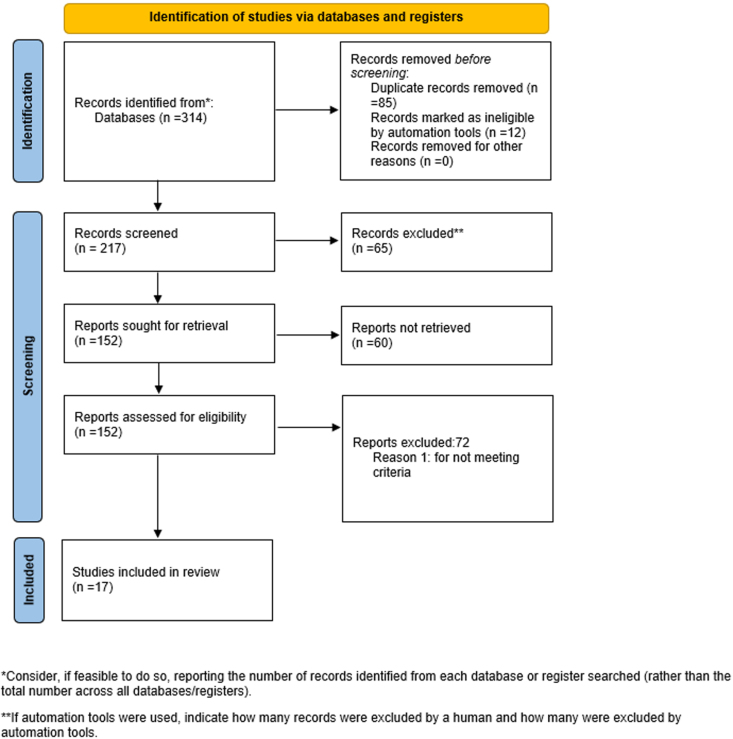
Flow diagram based on PRISMA-ScR guidelines. PRISMA-ScR = Preferred Reporting Items for Systematic Reviews and Meta-Analyses Extension for Scoping Reviews.

The systematic literature search initially identified 314 records from electronic databases and registers. After removing 85 duplicate records and 12 records marked as ineligible by automation tools, 217 records remained for title and abstract screening. Of these, 65 records were excluded, leaving 152 reports for further assessment. Additionally, 60 reports could not be retrieved. Consequently, 152 reports were assessed for full eligibility, of which 78 were excluded for the following reasons: inappropriate study design (n = 32, including reviews, editorials, and case reports), absence of relevant genetic data (n = 26), and outcomes not related to coronary IRS (n = 20) (Fig. 1). Ultimately, 9 studies (comprising 2 reports of included studies) were included in the review. These studies were analyzed following a predefined protocol, and relevant data were extracted systematically. Extracted data included author information, publication year, ethnicity of the study population, sample size, stent type, genotyping methods, genes of interest, restenosis occurrence, control group characteristics, *P* values, ORs, and CIs. This rigorous selection and data extraction process ensured a comprehensive and reproducible synthesis of evidence regarding genetic associations and risk factors for coronary IRS in the context of DESs.

#### 4.1.2. Coronary restenosis’ genetic

A comprehensive analysis of the included studies reveals a consistent and statistically significant association between several gene polymorphisms and the risk of coronary restenosis following DES implantation (Table 1). Notably, variants in the endothelial nitric oxide synthase (eNOS) gene, particularly the Glu298Asp (rs1799983) and T786C (rs2070744) polymorphisms, were found to be more prevalent among restenosis cases; for instance, the GT genotype of Glu298Asp appeared in 50% of cases versus 37% of controls, and the TT genotype of T786C was observed in 61% of cases compared to 54% of controls, both with significant *P* values (.003 and .01, respectively) and OR indicating elevated risk (OR 1.75 and 1.903). Similarly, the vascular endothelial growth factor (VEGF) −2549 I/D polymorphism showed a higher frequency of the II and ID genotypes in restenosis patients (II: 36%, ID: 40%) than in controls (II: 10%, ID: 27%), with a *P*-value of 0.029, underscoring its potential role in vascular healing. The AGT rs4762 T174M variant (TT genotype: 53% in cases vs 33% in controls, *P* = .005, OR 0.396, 95% CI: 0.170–0.430) and the REN rs41317140 (GG genotype: 62% in cases vs 56% in controls, *P* = .005, OR 4.062, 95% CI: 1.758–9.382) further highlight the involvement of the renin-angiotensin system (RAS) in restenosis susceptibility. Additionally, CYP2C19 loss-of-function alleles were strongly associated with restenosis risk, as individuals carrying 1 or 2 LOF alleles exhibited a markedly increased risk (OR 5.063, 95% CI: 1.610–15.921, *P* = .006). Polymorphisms in the adiponectin gene (APN +45T/G and +276G/T) and matrix metalloproteinase 3 (MMP-3 5A/6A) also demonstrated significant associations, with the TT genotype of APN +45T/G conferring an odds ratio of 2.874 (95% CI: 1.259–5.561) and the TT genotype of +276G/T yielding an OR of 1.368 (95% CI: 1.100–1.702). Collectively, these findings underscore the multifactorial genetic architecture underlying restenosis, with robust statistical evidence supporting the contribution of endothelial function, angiogenesis, vascular remodeling, and drug metabolism pathways. The observed associations, supported by significant *P* values and confidence intervals, not only reinforce the biological plausibility of these genetic risk factors but also suggest their potential utility as biomarkers for risk stratification and personalized management in patients undergoing coronary stenting (Table 2). A non-exhaustive list of papers included in the study (Fig. S1, Supplemental Digital Content 1, Tables S1 and S2, Supplemental Digital Content 4 and 5).

**Table 1:** Studies on coronary restenosis and stent insertion. .

	Author	Year	Ethnicity	Sample size	Stent	Genotyping	Gene/polymorphism	Restenosis group	Control group	*P* value	Odds ratio	Confidence interval
Vascular function and endothelial regulation	Asgarbeik, Saeedeha^[48]^	2022	White	120	DES	PCR	VEGF -2549 I/D	II 36%, ID 40%, DD 12%	II 10%, ID 27%, DD 26%	.029	–	–
	K. B. Timizheva	2020	White	113	DES	real time PCR	eNOS rs1799983 Glu298Asp	GG 45%, GT 50%, TT 5%	GG 53%, GT 37%, TT 10%	.003	–	–
	Ogorodova et al.	2017	White	484	DES	PCR	eNOS 894G/T	GG 26.7%, GT 46.7%, TT 26.6%	53.33%, 33.33%, 13.33%	.02	0.32	0.17–0.61
	Wen-ping Zeng	2017	Asia	425	DES	PCR multiplex	eNOS T786C	Tt 61%, Tc 33.3%, Cc 5.6%	Tt 76.8%, Tc 21%, Cc 2.2%	.03	1.903	1.162–3.116
	Ogorodova et al.	2017	White	484	DES	PCR	VNTR a/b	Bb 16.67%, Ab 43.33%, Aa 40.00%	42.67%, 36.00%, 21.33%	.02	0.27	1.29–4.70
Renin-angiotensin system	Madina Azova	2021	White	175	DES		AGT rs4762 T174M	TT 53%, TC 30%, CC 17%	TT 33%, TC 42%, CC 25%	.005	0.396	0.170–0.430
							REN rs41317140	GG 62%, AG 32%, AA 6%	GG 56%, AG 37%, AA 7%	.005	4.062	1.758–9.382
Extracellular matrix and fibrosis	Kamil Bujak	2021	White	657	—	TaqMan	CTGF (rs6918698) −945 G/C	GG 7.4%, CG 12.3%, CC 16.7%	GG 39.2%, GC 47.1%, CC 13.7%	.003	–	–
	Ji-Bing Du	2019	Asia	818	DES	PCR	MMP-3 5A/6A	5/5 0.3%, 5/6 25.5%, 6/6 74.2%	5/5 0.8%, 5/6 32.4%, 6/6 66.8%	.033	–	–
	Kalima B. Bogatyreva	2018	Russia	172	–	Rt PCR	TGB3 gene T1565C	X = 40, Cc 20.5%, Ct 27.5%, Tt 70%	X = 46, Cc 2%, Ct 26%, Tt 72%	–	–	–
Drug metabolism	Min Zhang	2020	Asia	111	DES	PCR	CYP2C19 LOF CYP2C19*2 & *3	No loss fun 11.8%, 1 LOF 40.7%, 2 LOF 6.1%	–	.006	5.063	1.610–15.921
Adiponectin and metabolic regulation	H.-C. LI	2019	Asia	150	–	TaqMan	APN + 45T/G	GG 25%, GT 32.14%, TT 42.86%	GG 8.20%, GT 36.03%, TT 55.74%	.754	2.874	1.259–5.561
	H.-C. LI	2019	Asia	150	–	TaqMan	+276G/T	GG 64.29%, GT 28.57%, TT 7.14%	40.98%, 37.71%, 21.31%	.047	1.368	1.100–1.702

– = data not reported, APN = adiponectin, CTGF = connective tissue growth factor, DES = drug-eluting stent, LOF = loss-of-function, PCR = polymerase chain reaction, VNTR = variable number tandem repeat.

**Table 2:** Association of genetic polymorphisms with coronary stent restenosis risk.

Gene/polymorphism	Genotype(s) associated with risk	Odds ratio (95% CI)	*P* value
eNOS Glu298Asp (rs1799983)	GT	1.75 (1.13–4.96)	.001
eNOS T786C (rs2070744)	Tt	1.903 (1.162–3.116)	.01
VEGF −2549 I/D	II, ID	–	.029
AGT rs4762 T174M	TT	0.396 (0.170–0.430)	.005
REN rs41317140	GG	4.062 (1.758–9.382)	.005
CYP2C19 LOF	1 or 2 LOF alleles	5.063 (1.610–15.921)	.006
APN +45T/G, +276G/T	TT	2.874 (1.259–5.561); 1.368 (1.100–1.702)	.754; .047
MMP-3 5A/6A	5/6, 6/6	1.109 (1.014–1.214)	.033

CI = confidence interval.

#### 4.1.3. Gene set enrichment analysis

This section presents the results of the gene set enrichment analysis, following the gene enrichment methodology previously described.

#### 4.1.4. Collecting data

**Figure 2.**
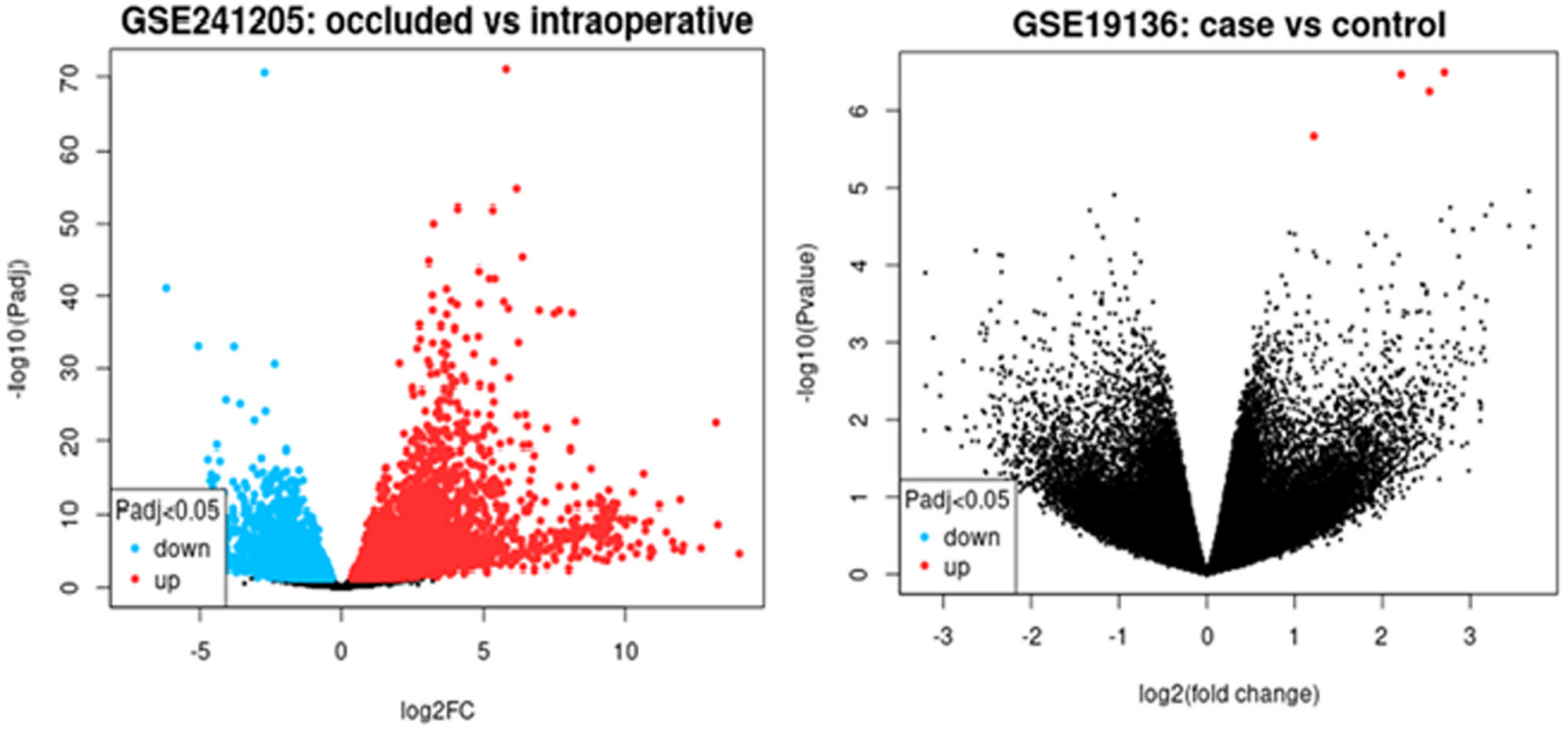
Illustrates the GEO2R analysis results, comparing case and control studies. The analysis on the right corresponds to the 2-group case and control study from GSE241205, while the analysis on the left represents GSE19136. GEO2R = analytical tool of Gene Expression Omnibus database.

The analysis utilized 2 expression profile datasets from the GEO database: GSE19136 involved human left internal mammary artery segments treated with either a paclitaxel-eluting stent or a BMS, alongside a control group. Each group comprised 4 biological replicates, derived from patients 1, 2, 4, and 5. GSE182225 included 3 independent experiments for each group, with different donors used in each experiment. Both datasets provided a robust foundation for downstream enrichment analysis (Fig. 2).

## 5. Functional enrichment analysis of identified genes

### 5.1. Selection and cleaning process for gene inclusion in enrichment analysis

To ensure robust and biologically meaningful results in the enrichment step, a systematic selection and cleaning process was applied to the gene expression data:

- Initial filtering: Raw expression data from the GSE241205 dataset (occluded vs intraoperative) underwent quality control to remove low-quality samples and genes with minimal or inconsistent expression across replicates.
- Normalization: Data was normalized to account for sequencing depth and technical variation, enabling accurate comparison between conditions.
- Statistical testing: Differential expression analysis was performed, identifying genes with adjusted *P* values (*P* adj) <.05 to control false discovery rate.
- Stringent fold change cutoff: Only genes with substantial expression changes (|log2FC| > 5) were retained, focusing on those with the most pronounced biological effects.
- Artifact exclusion: Genes with extreme expression changes but lacking biological plausibility or consistency across replicates were flagged as potential artifacts and excluded from downstream analyses.
- Annotation and validation: The resulting list of DEGs was annotated using current gene databases, and candidates for further validation (e.g., quantitative polymerase chain reaction) were prioritized based on their relevance to ischemia, hypoxia, or inflammation.

### 5.2. Differentially expressed genes and their implications

Analysis of the volcano plot revealed a focused subset of 10 to 15 DEGs with high statistical significance and large fold changes. Approximately 5 genes were markedly downregulated (log2 FC < −5) in occluded samples, while 5 to 10 genes were sharply upregulated (log2 FC > 5). These pronounced changes likely reflect genes specifically activated or suppressed in response to acute ischemia or cellular stress, rather than broad, nonspecific alterations. Their specificity points to roles in critical adaptive or pathological pathways.

### 5.3. Enriched GO terms

**Figure 3.**
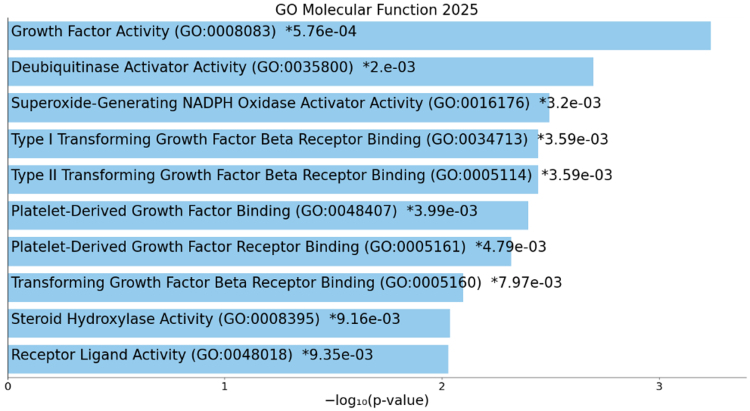
Pathway generated using the ClueGO plug-in in Cytoscape software. GO = gene ontology.

GO enrichment analysis highlighted several molecular functions with significant overrepresentation: Growth Factor Activity (GO:0008083): Encompasses genes encoding proteins like VEGF, FGF, and platelet-derived growth factors (PDGF), which drive cell proliferation, angiogenesis, and tissue repair key processes during ischemic injury and recovery. Transforming growth factor beta 1 (TGFβ/TGFB1) receptor binding (GO:0005160): TGFβ signaling modulates inflammation, fibrosis, and immune responses, with altered activity linked to tissue remodeling and vascular pathology. PDGF binding (GO:0048407): PDGF is crucial for vascular stability and repair, influencing smooth muscle cell (SMC) migration and proliferation. Superoxide-Generating Nicotinamide Adenine Dinucleotide Phosphate Oxidase Activator Activity (GO:0016176): Genes in this category regulate oxidative stress, a hallmark of ischemic and reperfusion injury. Deubiquitinate Activator Activity (GO:0035800): Involvement in protein stability and turnover, potentially modulating stress responses and cell survival. These enriched terms point to a coordinated response involving growth factor signaling, oxidative stress regulation, and protein homeostasis, all of which are central to the biological adaptation to vascular occlusion and reperfusion (Fig. 3).

**Figure 4.**
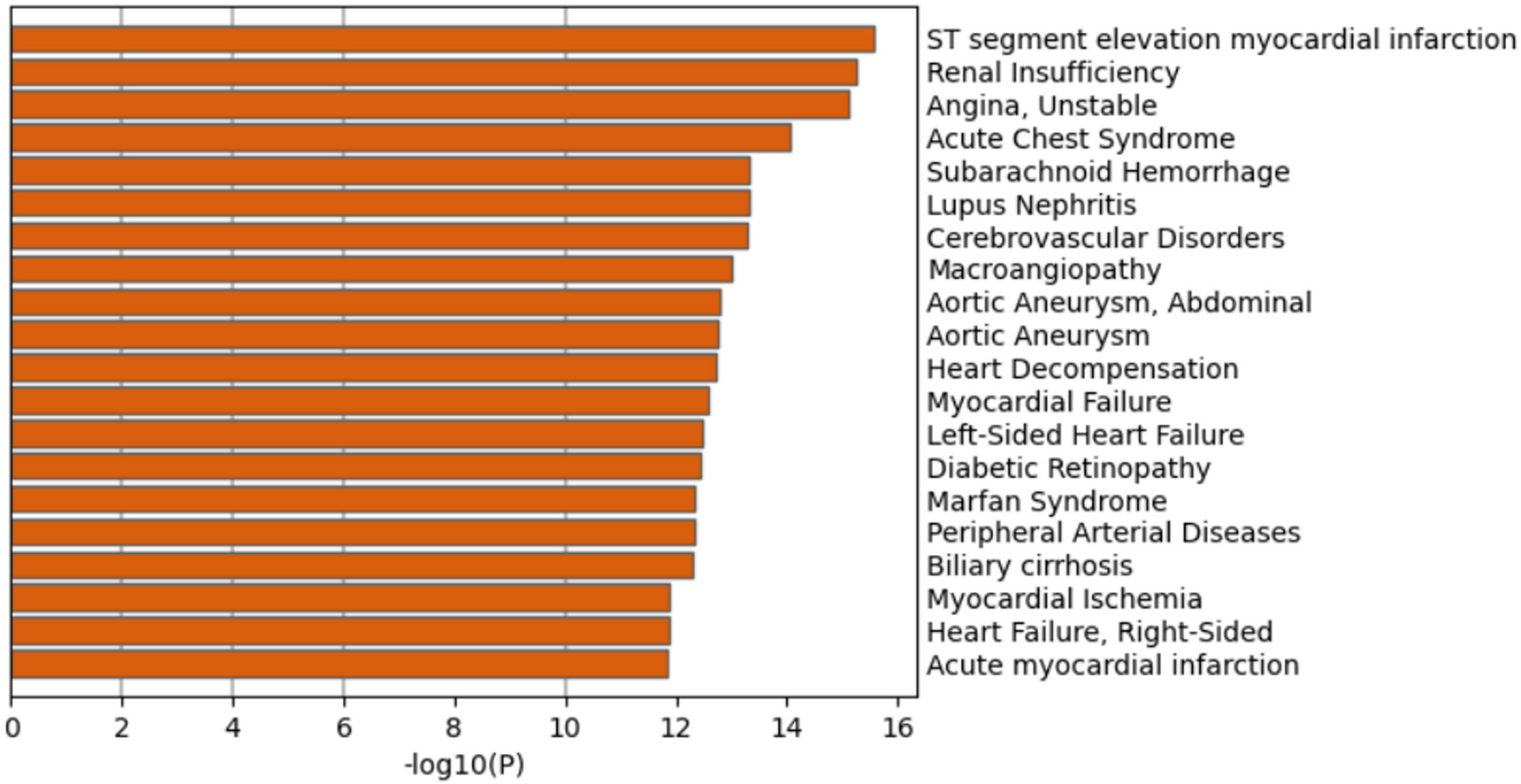
Summary of enrichment analysis in DisGeNET.

The functional enrichment network generated using Metascape revealed a highly interconnected landscape of biological processes associated with the analyzed gene set. Each node represents a significantly enriched term, while edges indicate functional similarity based on shared genes, resulting in the formation of distinct clusters of related processes. Prominent clusters included endocrine-related processes, regulation of kidney and lung development, positive regulation of cellular component biogenesis, and cellular responses to xenobiotic stimuli and bacterial challenge. Notably, processes related to the negative regulation of SMC proliferation were also identified, highlighting mechanisms relevant to vascular remodeling. The dense connectivity observed across clusters suggests a high degree of functional overlap and coordination among the enriched pathways, indicating that the selected genes participate in multiple, interrelated biological processes (Fig. 4).

### 5.4. Key pathways and their biological significance

The pathway analysis identified 3 principal nodes: angiotensin system: angiotensinogen is converted to angiotensin peptides (e.g., angiotensin II) via angiotensin-converting enzyme (ACE). Angiotensin II is a potent regulator of blood pressure, fluid balance, and vascular tone, with dysregulation implicated in hypertension and cardiovascular disease. ACE inhibitor pathway: Pharmacological inhibition of ACE prevents the formation of angiotensin II, reducing vasoconstriction and lowering blood pressure. This pathway is a major therapeutic target in hypertension and heart failure management. Phosphoenolpyruvate carboxykinase: a key enzyme in gluconeogenesis, phosphoenolpyruvate carboxykinase links carbon metabolism to energy homeostasis. Its involvement suggests metabolic adaptation in response to ischemic stress, potentially influencing cellular survival and function under hypoxic conditions at context of key genes and pathways.

### 5.5. Pathways and GO terms significantly enriched

**Figure 5.**
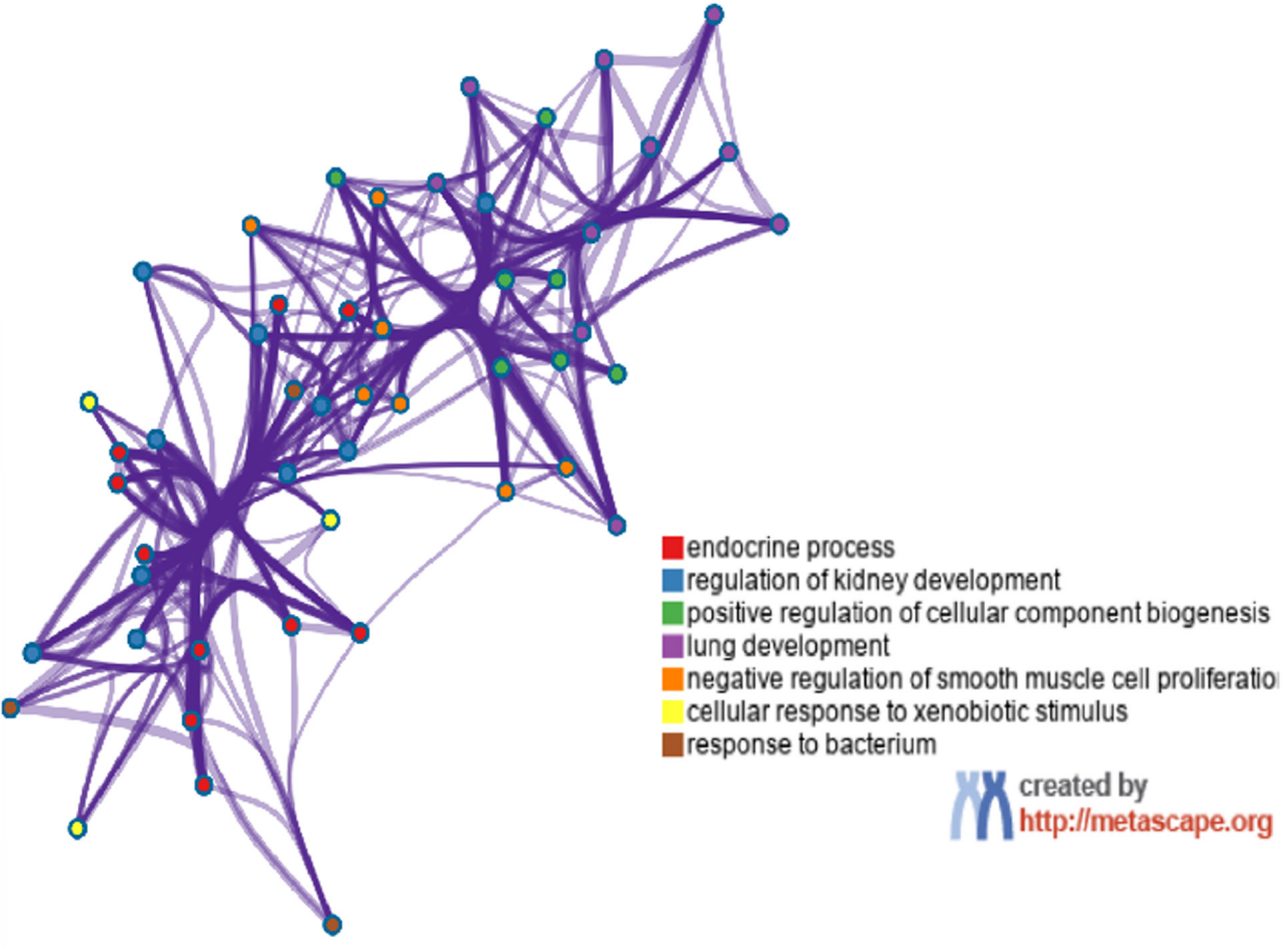
Network of enriched terms colored by cluster ID, where nodes that share the same cluster ID are typically close to each other. Generated by metascape.

Analysis of the GSE241205 dataset (comparing occluded to intraoperative samples) revealed a focused group of 10 to 15 DEGs with both high statistical significance (*P* adj < .05) and substantial fold changes (|log2 FC| > 5). Within this subset, about 5 genes were strongly downregulated (log2 FC < −5) in occluded samples, while another 5 to 10 genes were sharply upregulated (log2 FC > 5). These pronounced changes suggest that only a select group of genes undergo significant transcriptional shifts, rather than a broad, nonspecific response. Such extreme DEGs likely represent genes involved in key biological pathways activated by ischemia or cellular stress, although the possibility of technical artifacts cannot be excluded. As a result, further validation such as quantitative polymerase chain reaction and additional pathway analyses, particularly those focusing on hypoxia and inflammation, is essential to confirm these findings and clarify their mechanistic relevance (Fig. 5).

**Figure 6.**
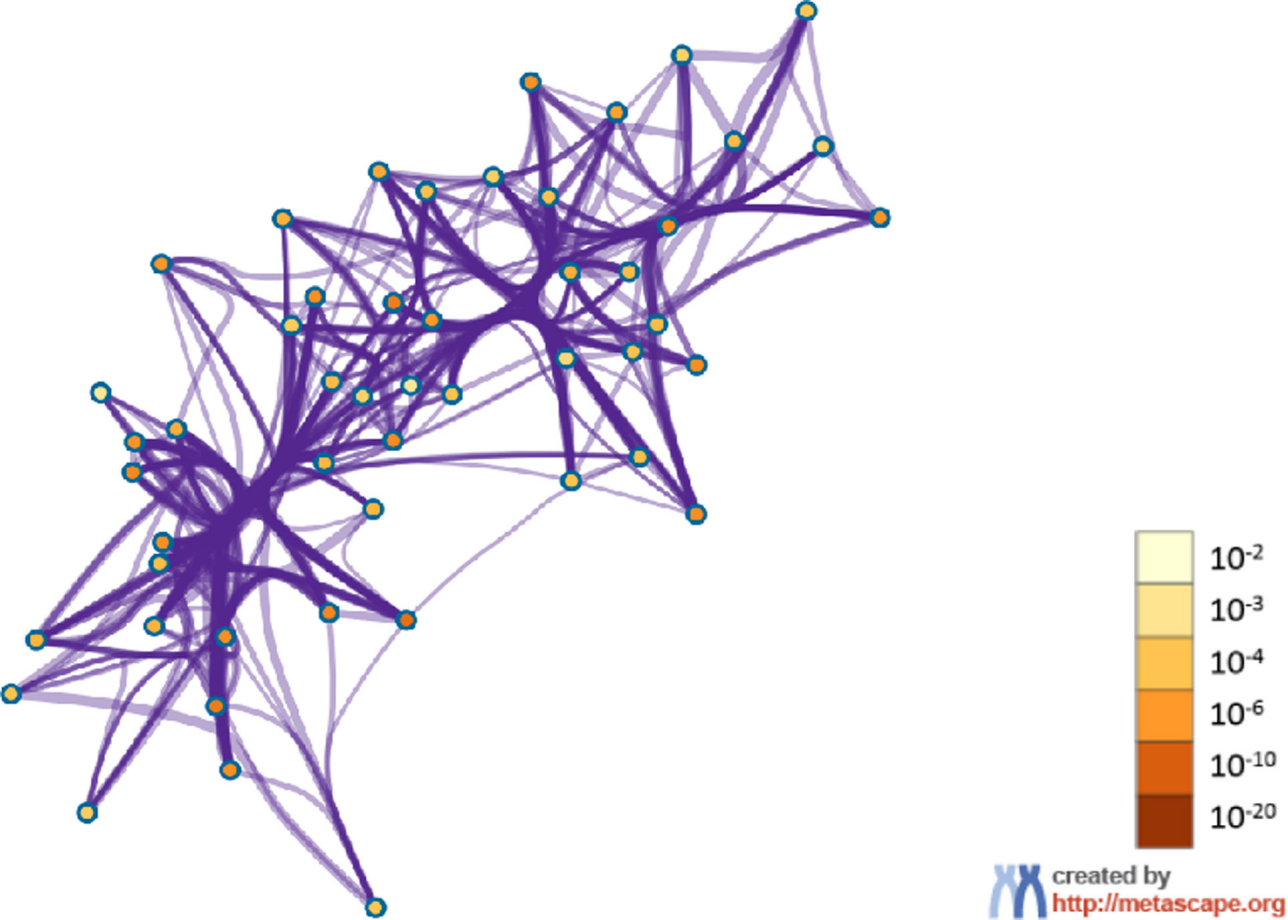
Network of enriched terms colored by *P* value, where terms containing more genes tend to have a more significant *P* value.

The disease enrichment analysis performed using the DisGeNET database revealed that the selected gene set is significantly associated with a broad spectrum of cardiovascular and systemic diseases. The most strongly enriched conditions included ST-segment elevation myocardial infarction, acute myocardial infarction, myocardial ischemia, and heart failure, all showing high statistical significance (−log10 *P* values >10) (Table 3). In addition, several vascular disorders, such as aortic aneurysm, peripheral arterial disease, macroangiopathy, and cerebrovascular diseases, were prominently represented. Notably, inflammatory and metabolic conditions, including lupus nephritis, renal insufficiency, and diabetic retinopathy, were also identified among the enriched terms (Fig. 6). Overall, these findings indicate that the analyzed genes are predominantly implicated in cardiovascular, vascular remodeling, and inflammation-related disease processes. Additional evidence weights scoring for the studies is included in the supplementary files (Fig. 2, Supplemental Digital Content 2, Tables S3–S5, Supplemental Digital Content 3, 6 and 7).

**Table 3:** Enrichment analysis using DisGeNet.

GO	Description	Count	%	Log10 (*P*)	Log10 (*q*)
C1536220	ST-segment elevation myocardial infarction	8	89.00	−16.00	−11.00
C1565489	Renal insufficiency	9	100.00	−15.00	−11.00
C0002965	Angina, unstable	7	78.00	−15.00	−11.00
C0742343	Acute chest syndrome	8	89.00	−14.00	−10.00
C0038525	Subarachnoid hemorrhage	8	89.00	−13.00	−9.60
C0024143	Lupus nephritis	8	89.00	−13.00	−9.60
C0007820	Cerebrovascular disorders	7	78.00	−13.00	−9.60
C1096293	Macroangiopathy	5	56.00	−13.00	−9.40
C0162871	Aortic aneurysm, abdominal	8	89.00	−13.00	−9.20
C0003486	Aortic aneurysm	7	78.00	−13.00	−9.20
C1961112	Heart decompensation	6	67.00	−13.00	−9.20
C1959583	Myocardial failure	6	67.00	−13.00	−9.10
C0023212	Left-sided heart failure	6	67.00	−12.00	−9.10
C0011884	Diabetic retinopathy	8	89.00	−12.00	−9.10
C0024796	Marfan syndrome	6	67.00	−12.00	−9.00
C1704436	Peripheral arterial diseases	7	78.00	−12.00	−9.00
C0023892	Biliary cirrhosis	6	67.00	−12.00	−9.00
C0151744	Myocardial ischemia	8	89.00	−12.00	−8.60
C0235527	Heart failure, right-sided	6	67.00	−12.00	−8.60
C0155626	Acute myocardial infarction	8	89.00	−12.00	−8.60

GO = gene ontology.

### 5.6. Genes and pathways implicated in ISR

The combined results from gene expression analysis and systematic review highlight a consistent set of genes and pathways central to the development of ISR following DES implantation. Key genes implicated include those involved in growth factor signaling (such as VEGFA and PDGFB), ECM regulation (notably MMPs), and inflammatory pathways (including interleukin-6 and tumor necrosis factor-α [TNF-α]). Differential expression analyses further identified genes associated with oxidative stress regulation (e.g., Nicotinamide adenine dinucleotide phosphate oxidase activators), protein stability (deubiquitinase activators), and vascular remodeling. The most significantly enriched pathways encompass growth factor activity, TGFβ and PDGF receptor signaling, and the angiotensin system, all of which orchestrate processes like cell proliferation, migration, tissue repair, and immune modulation. Collectively, these findings underscore that ISR is driven by a coordinated response involving vascular injury repair, inflammation, oxidative stress, and metabolic adaptation, with specific genes and pathways serving as potential biomarkers and therapeutic targets for improved patient outcomes (Fig. 6).

## 6. Discussion

Our analysis revealed that coronary stent restenosis is associated with the involvement of multiple biological pathways, as identified through bioinformatic tools, including pathway and ontology enrichment analysis. Using platforms such as Enrich and visualization tools like Apyter, we mapped genes with significant log fold changes (Log FC) to enrich pathways. A substantial number of these pathways were related to inflammatory responses and metalloprotein activity, highlighting the critical roles of immune system dysregulation and ECM remodeling in the restenosis process.

### 6.1. ECM remodelling and growth factor signalling

Genes involved in ECM remodeling and growth factor signaling constitute a prominent mechanistic axis implicated in ISR. TGFβ plays a central role in vascular repair, SMC activation, and collagen deposition.^[42,43]^ Experimental models have demonstrated that early inhibition of TGFβ signaling reduces intimal hyperplasia, whereas sustained TGFβ activity promotes matrix accumulation and influences vessel remodeling.^[44]^ Clinical studies examining TGFB1 polymorphisms have yielded heterogeneous results, with reported effects varying by population, stent type, and genetic variant. While some associations suggest the modulation of neointimal growth, much of the available evidence is derived from BMS-treated populations. Osadnik et al reported that carriers of the −869T allele exhibit reduced neointimal hyperplasia following BMS implantation.^[45]^ Similarly, analyses of the TGFB1 29C>T polymorphism have indicated that patients with the A/A genotype experience lower late lumen loss and lower rates of IRS than patients with other haplotypes.^[46]^

Connective tissue growth factor, a downstream mediator of TGFβ signaling, has also been implicated in fibrotic responses and restenosis-related outcomes. Associations between Connective tissue growth factor polymorphisms and target lesion revascularization support a contributory role of profibrotic signaling pathways.^[47]^ However, DES-specific data remains limited, underscoring the need for further studies focused on contemporary stent platforms.

Angiogenic signaling through VEGF, which is upregulated in response to vascular injury and inflammation, has been explored. VEGF promotes neointimal formation through mechanisms involving SMC activation, monocyte recruitment, and adventitial angiogenesis.^[48]^ Meta-analyses have suggested that VEGF polymorphisms exhibit ethnicity-dependent associations, with the 1154G>A variant linked to coronary disease in Caucasian populations and the +936C>T variant associated with susceptibility among Asian.^[45,49,50]^ Overall, evidence supporting a role for VEGF variants in ISR remains suggestive but insufficiently robust, particularly in DES-treated populations.^[51–53]^

Inflammatory signaling through TNF-α, which has been identified as a potential susceptibility factor for restenosis in the GENDER study, has also been investigated. Although certain promoter polymorphisms, such as G308A and G238A, have been associated with coronary heart disease in some reports (20, 33), large-scale investigations have yielded less consistent findings in the context of restenosis. In a study involving 1850 stented patients, Koch et al reported no significant association between TNF-α promoter variants (−863C/A and −308G/A) and restenosis risk^[54,55]^

### 6.2. Genes of renin-angiotensin aldosterone system

The RAS plays a central mechanistic role in ISR by promoting vascular remodeling and inflammation.^[56,57]^ Angiotensin II, the primary effector of RAS, stimulates VSMC.^[58]^ proliferation, migration, and extracellular matrix production, leading to neointimal hyperplasia and vessel wall thickening key features of ISR. RAS activation also enhances oxidative stress and upregulates inflammatory mediators such as monocyte chemoattractant protein-1,^[59,60]^ which recruits immune and progenitor cells to the site of vascular injury; these cells can differentiate into VSMCs, further driving neointimal growth.^[59,61]^ Bioinformatic analyses of ISR tissues and blood samples reveal by pathway enrichment, consistently highlights the mitogen-activated protein kinase signaling and cytokine-cytokine receptor interaction pathways as major downstream targets of angiotensin II,^[58,62]^ linking RAS activity to the broader inflammatory response in ISR. Systematic reviews confirm that RAS activation amplifies these inflammatory pathways, and clinical studies show that RAS inhibition; particularly with angiotensin II type 1 receptor blockers and ACE inhibitors can reduce neointimal formation and ISR risk by up to 30%.^[63]^ However, the multifactorial nature of ISR means that no single marker or pathway reliably predicts risk, underscoring the need for network-based approaches to improve risk stratification and personalized therapy.^[64]^

### 6.3. Endothelial sysfunction nitric oxide synthase (eNOS) gene

Among the biological pathways identified, endothelial dysfunction and dysregulation of vascular homeostasis emerged as the most consistently represented mechanistic axes underlying genetic susceptibility to IRS.^[65–67]^ Preservation of endothelial integrity is essential for effective vascular healing after stent implantation, and impaired or delayed endothelialisation has been widely recognized as a key contributor to restenosis in the DES era.^[68–70]^ Endothelial nitric oxide synthase plays a central role in regulating nitric oxide bioavailability, which exerts vasodilatory, anti-inflammatory, antithrombotic, and antiproliferative effects within the vascular wall. Bioinformatic analyses and systemic research have identified eNOS as a susceptibility gene for various cardiovascular diseases, including ISR. Functional nitric oxide synthase 3 polymorphisms, including − 786T > C and G894T (Glu298Asp), influence enzyme expression or activity and have been previously linked to susceptibility to cardiovascular disease.^[71,72]^ In the context of ISR, several studies have reported associations between nitric oxide synthase 3 variants and restenosis risk across diverse populations.^[73]^ The evidence is most consistent for the −786T>C polymorphism, particularly in Asian cohorts, where larger sample sizes and repeated observations support a contributory role.^[71]^ Meta-analyses have further suggested that the G894T variant may be associated with increased ISR risk in both Caucasian and Asian populations.^[70]^ Nonetheless, the variability in effect estimates and population-specific findings highlighted the need for adequately powered, multiethnic studies to more precisely define the magnitude and clinical relevance of these associations^[74,75]^

### 6.4. MMP genes

MMPs, a family of zinc- and calcium-dependent endopeptidases are central to the dynamic turnover of the ECM during vascular remodeling in ISR.^[76]^ These enzymes are classified by substrate specificity into collagenases (MMP-1, -8, -13, -18), gelatinases (MMP-2, -9), stromelysins (MMP-3, -10, -11, -19, -20), and membrane-type MMPs (MMP-14, -15, -16, -17). In ISR, especially after DES placement, is characterized by proteoglycan-rich neointimal hyperplasia and reduced SMC content.^[74,77]^ Bioinformatic tools play a crucial role in unraveling the involvement of MMPs in ISR. High-throughput gene expression profiling identifies differentially expressed MMP genes in ISR tissues and blood samples, revealing regulatory patterns associated with neointimal hyperplasia. PPI network analyses position MMPs as central nodes linked to inflammatory cytokines and growth factors, clarifying their coordination of ECM remodeling and inflammation. Pathway enrichment analyses, using resources like KEGG and Reactome, highlight the influence of MMP expression changes on ECM organization, inflammatory signaling, and cell migration. Additionally, computational analyses of genetic polymorphisms – such as the MMP-3 (5A/6A) variant integrate data from genome-wide association studies and meta-analyses to assess ISR susceptibility. Key findings indicate that neointimal hyperplasia is marked by decreased MMP-2 and MMP-9 expression and increased collagen deposition, while elevated plasma levels of MMP-3 and MMP-9 are predictive biomarkers for ISR, particularly in BMS recipients.^[76,78]^ The MMP-3 (5A/6A) promoter polymorphism is associated with restenosis risk, with the 6A/6A genotype conferring higher susceptibility and the 5A allele being potentially protective in some populations.^[75]^ Collectively, these bioinformatic and systemic approaches underscore the multifaceted role of MMPs as both mechanistic contributors and potential biomarkers in the risk and pathogenesis of restenosis.

### 6.5. Drug metabolism and platelet response cytochrome P450 2C 19

Genetic variability influencing antiplatelet drug metabolism represents an additional biological pathway that may indirectly contribute to ISR, particularly in patients treated with DESs who receive clopidogrel-based dual-antiplatelet therapy.^[79]^ The CYP2C19 gene encodes a key hepatic enzyme responsible for the bioactivation of clopidogrel, and loss-of-function alleles (most notably, CYP2C19 *2 and *3) are consistently associated with reduced platelet inhibition following percutaneous coronary intervention.^[80]^ Within the context of ISR, several studies have reported associations between CYP2C19 loss-of-function variants and increased restenosis risk.^[81]^ with the strongest and most reproducible signals observed in Asian populations, where the prevalence of these alleles is relatively high.^[82–84]^ From a mechanistic perspective, impaired clopidogrel activation leads to increase on-treatment platelet reactivity, which may exacerbate local inflammatory signaling, endothelial dysfunction, and thrombo-inflammatory responses at the stented segment.^[85]^ Although pharmacogenetic trials have demonstrated that genotype-guided antiplatelet strategies improve overall post-PCI outcomes, evidence directly linking such approaches to reduced ISR remains limited. Conversely, gain-of-function variants, such as CYP2C19 *17, have been associated with enhanced clopidogrel responsiveness and more favorable procedural outcomes, including lower rates of target lesion revascularization in some cohorts.^[80,81,86]^ In addition to drug metabolism, variations in platelet receptor signaling may further modulate vascular healing. The P2Y12 receptor, the pharmacological target of clopidogrel, plays a central role in platelet activation and aggregation. While functional P2RY12 polymorphisms have been shown to influence platelet reactivity in pharmacodynamic studies, their direct association with ISR has not been consistently demonstrated across populations.^[79,87,88]^

## 7. Limitation

This study has several limitations that should be considered when interpreting the findings. First, the review combines elements of a systematic review with a scoping and bioinformatic approach. While this hybrid design allowed for both comprehensive literature synthesis and in-depth functional pathway analysis, it may limit direct comparability with purely systematic reviews. Second, the protocol for this study was not prospectively registered in PROSPERO. Although the absence of registration does not affect the validity of the results, prospective registration is increasingly recommended for transparency and to minimize potential reporting bias. Additionally, most included genetic association studies were heterogeneous in terms of stent types (BMS vs DES), patient populations, follow-up durations, and ethnic backgrounds, which may have influenced the consistency of findings. The bioinformatic analyses were based on publicly available datasets, which may carry inherent limitations related to sample size, platform differences, and batch effects. Finally, although we focused on the most frequently reported genes and pathways, emerging or less-studied genetic variants may have been underrepresented.

Despite these limitations, this work provides a comprehensive integration of genetic evidence and functional bioinformatics that highlights key mechanisms in coronary stent restenosis and supports future precision medicine approaches.

## 8. Conclusion

In conclusion, this study offers a thorough molecular characterization of coronary stent restenosis, emphasizing the pivotal roles of inflammation, extracellular matrix remodeling, and the renin-angiotensin system in its pathogenesis. By integrating systematic review with enrichment-based bioinformatic analysis, we have identified gene networks and pathways that not only deepen our understanding of restenosis mechanisms but also highlight promising biomarkers and therapeutic targets. These insights contribute to the evolving landscape of precision medicine, paving the way for more individualized risk assessment, prevention, and treatment strategies to enhance outcomes for patients receiving DESs.

Supplemental Digital Content “Tables S4 and S5, Supplemental Digital Content 3” are available for this article.

## Acknowledgment

The authors would like to thank the members of the Laboratory of Medical Genetics at *Ibn Rochd* University Hospital for their valuable support and technical assistance throughout this study. We are also grateful to the Department of Cardiology at *Ibn Rochd* University Hospital for their collaboration and facilitation in this research.

## Author contributions

**Conceptualization:** Rajaa EL Mansouri, Rachida Habbal.

**Data curation:** Hind Dehbi.

**Resources:** Hind Dehbi.

**Supervision:** Rachida Habbal.

**Writing – original draft:** Rajaa EL Mansouri.

**Writing – review & editing:** Rachida Habbal.

## Correction

This article was originally published with incorrect wording in the title and the How to Cite section. The incorrect word “testenosis” has now been corrected in the online version to “restenosis.”














