# Molecular profiling of coronary stent restenosis. A systematic review and functional analysis of implicated genes: Erratum

**DOI:** 10.1097/MD.0000000000049960

**Published:** 2026-07-24

**Authors:** Rajaa EL Mansouri, Rachida Habbal, Hind Dehbi

The article “Molecular profiling of coronary stent restenosis. A systematic review and functional analysis of implicated genes”,^[1]^ which appears in Volume 105, Issue 26 of *Medicine*, was originally published with incorrect word in the title.

The incorrect title has been now updated online from “Molecular profiling of coronary stent testenosis. A systematic review and functional analysis of implicated genes” to “Molecular profiling of coronary stent restenosis. A systematic review and functional analysis of implicated genes.”
