# Supplementary figures and images for: Molecular profiling of coronary stent restenosis: A systematic review and functional analysis of implicated genes
Source: Medicine (Baltimore). 2026 Jun 26;105(26):e49455. doi: 10.1097/MD.0000000000049455 (PMC13313781; doi:10.1097/MD.0000000000049455)

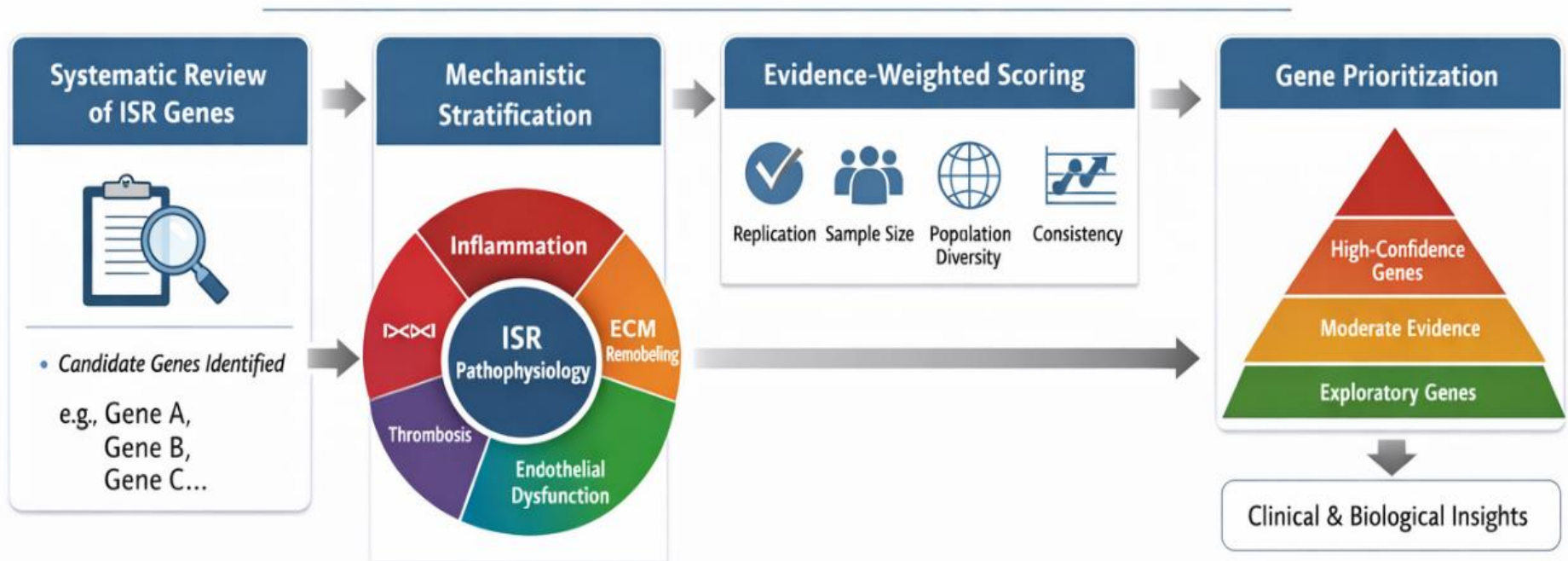

Supplementary figure 1: integrative bioinformatics framework for gene prioritization.

Supplement: Supplementary file 1 [file medi-105-e49455-s001.pdf]
