# Supplementary material for: Molecular profiling of coronary stent restenosis: A systematic review and functional analysis of implicated genes
Source: Medicine (Baltimore). 2026 Jun 26;105(26):e49455. doi: 10.1097/MD.0000000000049455 (PMC13313781; doi:10.1097/MD.0000000000049455)
Supplement: Supplementary file 2 [file medi-105-e49455-s002.pdf]

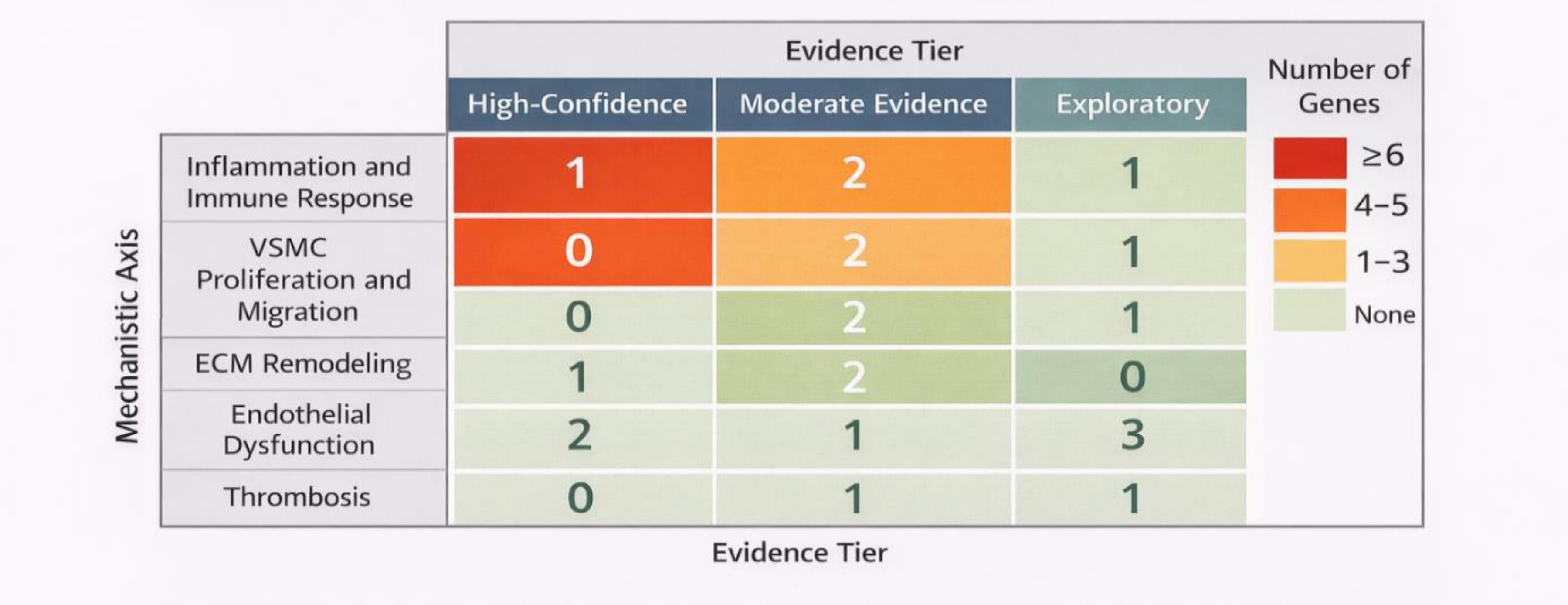

Supplementary figure 2 : Mechanistic pathways involved in restenosis, distributed by evidence tier.
