# Supplementary material for: Molecular profiling of coronary stent restenosis: A systematic review and functional analysis of implicated genes
Source: Medicine (Baltimore). 2026 Jun 26;105(26):e49455. doi: 10.1097/MD.0000000000049455 (PMC13313781; doi:10.1097/MD.0000000000049455)
Supplement: Supplementary file 3 [file medi-105-e49455-s003.docx]

**Mechanistic Stratification of ISR-Associated Genes**

Following mechanistic stratification, ISR-associated genes were distributed across five predefined biological axes reflecting established restenosis pathophysiology. Genes related to endothelial dysfunction and vascular regulation constitute the most represented category, primarily driven by multiple polymorphisms within the *NOS3 (eNOS)* gene and the inclusion of *VEGF*. Genes involved in extracellular matrix remodeling and fibrosis, including *CTGF*, *MMP3*, and *TGFB3*, represented the second most frequent mechanistic group. Additional genes were assigned to the renin–angiotensin system, drug metabolism, and metabolic/adipokine regulation axes. Several genes were assigned to more than one mechanistic category, reflecting overlapping biological functions across endothelial regulation, inflammatory signaling, and structural remodeling processes. (table III)

*Title: Mechanistic Stratification of ISR-Associated Genes*

*Supplementary Table 4: criterion for mechanistic stratification of ISR associated genes.*

| **Criterion** | **Points** |
| --- | --- |
| **≥2 independent studies** | **2** |
| **≥1 study with sample size >500** | **2** |
| **Replication across populations** | **2** |
| **Consistent direction of association** | **1** |
| **DES-specific analysis** | **1** |
