# Supplementary material for: Molecular profiling of coronary stent restenosis: A systematic review and functional analysis of implicated genes
Source: Medicine (Baltimore). 2026 Jun 26;105(26):e49455. doi: 10.1097/MD.0000000000049455 (PMC13313781; doi:10.1097/MD.0000000000049455)
Supplement: Supplementary file 4 [file medi-105-e49455-s004.docx]

Title Studies included in the study.

Supplementary Table 1: Studies included in the study.

| **Study (Year)** | **Population / Era** | **Stent Platform** | **ISR Definition & Follow-up** | **Primary Endpoint** | **Gene / Polymorphism** | **Genetic Model** | **Adjusted Effect (OR, 95% CI)** | **Covariates Adjusted** | **Risk-of-Bias Notes** |
| --- | --- | --- | --- | --- | --- | --- | --- | --- | --- |
| Tabatabaei & al.[70] | Iranian; DES era | DES (2nd gen) | ≥50% angiographic stenosis; 6–12 mo | Binary ISR | eNOS −786 T>C | Recessive | 1.90 (NR) | Age, sex, DM | Small sample; limited covariate adjustment |
| Zeng & al.[86] | Chinese Han; DES era | DES (2nd gen) | ≥50% angiographic stenosis; 12 mo | Binary ISR | eNOS −786 T>C | Dominant | 1.75 (NR) | Age, sex, DM, HTN | Single &hnicity |
| Zhou & al. [88] | Mixed Asian/Caucasian | DES/BMS | Angiographic ISR; ≤12 mo | Binary ISR | eNOS Glu298Asp | Additive | 1.68 (NR) | Variable | M&a-analysis h&erogeneity |
| Ogorodova & al.[51] | Russian; mixed era | DES/BMS | ≥50% stenosis; 6 mo | Binary ISR | eNOS G894T | Dominant | 2.10 (NR) | NR | Limited adjustment |
| Azova & al. [5] | Kazakh; DES era | DES | ≥50% stenosis; 12 mo | Binary ISR | AGT M235T | Recessive | 2.40 (NR) | Age, sex, DM | Moderate sample size |
| Zhou & al.[88] | Caucasian | DES/BMS | Angiographic ISR; 6–12 mo | Binary ISR | AGT M235T | Dominant | 1.85 (NR) | Age, sex | &hnic-specific effect |
| Xu & al.[85] | Chinese | DES | ISR vs no ISR | Binary ISR | AT1R A1166C | Additive | NS | Age, sex | Null finding |
| Shuanzhou & al.[21] | Asian | DES/BMS | ≥50% stenosis; ≤12 mo | Binary ISR | MMP3 5A/6A | Dominant | 1.11 (1.01–1.21) | Age, sex | Single SNP |
| Du & al. | Chinese Han | BMS/PTCA | Angiographic restenosis | Binary restenosis | MMP3 5A/6A | Recessive | 1.34 (NR) | Age, sex | Short follow-up |
| Zhang & al.[87] | Asian; DES era | DES | ≥50% stenosis; 12 mo | Binary ISR | CYP2C19 LOF | Dominant | 1.19 (NR) | Age, sex, DM | Drug adherences unclear |
| Ozawa & al.[54] | Japanese | DES | Clinical ISR / TLR | TLR | CYP2C19 metabolizer | Categorical | Reduced events | Age, sex | Clinical endpoint |
| Monraats & al. [40] | European | BMS | Clinical restenosis | TLR / ISR | IL-10 variants | Haplotype | Signal only | Multivariable | BMS-specific |
| Koch & al.[40] | European | BMS | Angiographic ISR | Binary ISR | TNFα −308G/A | Additive | NS | Age, sex | Large sample, null |
| Bujak & al.[12] | European | BMS | Target lesion revascularization | TLR | CTGF polymorphism | Dominant | Signal | Age, sex | Clinical endpoint |
