# Supplementary material for: Molecular profiling of coronary stent restenosis: A systematic review and functional analysis of implicated genes
Source: Medicine (Baltimore). 2026 Jun 26;105(26):e49455. doi: 10.1097/MD.0000000000049455 (PMC13313781; doi:10.1097/MD.0000000000049455)
Supplement: Supplementary file 5 [file medi-105-e49455-s005.docx]

*Title: summary of characteristics and extracted genetic data*

*Supplementary Table 2: summary of characteristics and extracted genetic data*

| **Author** | **Year** | **ethnicity / Region** | **Sample size** | **Stent type** | **Genotyping m&hod** | **Gene** | **Polymorphism** | **ISR outcome definition*** |
| --- | --- | --- | --- | --- | --- | --- | --- | --- |
| Asgarbeik & al.[4] | 2022 | White | 120 | DES | PCR | VEGF | −2549 I/D | Angiographic ISR |
| Timizheva & al.[74] | 2020 | White | 113 | DES | Real-time PCR | NOS3 (eNOS) | rs1799983 (Glu298Asp) | Angiographic ISR |
| Ogorodova & al (1).[51] | 2017 | White | 484 | DES | PCR | NOS3 (eNOS) | 894G/T | Angiographic ISR |
| Zeng H& al.[5] | 2017 | Asian | 425 | DES | Multiplex PCR | NOS3 (eNOS) | T786C | Angiographic ISR |
| Ogorodova & al(2).[51] | 2017 | White | 484 | DES | PCR | NOS3 (eNOS) | VNTR a/b | Angiographic ISR |
| Azova & al (1).[5] | 2021 | White | 175 | DES | Not specified | A[47]GT | rs4762 (T174M) | Angiographic ISR |
| Azova & al.[5] | 2021 | White | 175 | DES | Not specified | REN | rs41317140 | Angiographic ISR |
| Bujak & al.[12] | 2021 | White | 657 | Not specified | TaqMan | CTGF | rs6918698 (−945 G/C) | Angiographic ISR |
| Du & al.[21] | 2019 | Asian | 818 | DES | PCR | MMP3 | 5A/6A | Angiographic ISR |
| Deep&hy & al.[20] | 2018 | Russian | 172 | Not specified | RT-PCR | TGFB3 | T1565C | Angiographic ISR |
| Zhang & al.[87] | 2020 | Asian | 111 | DES | PCR | CYP2C19 | *2, *3 (LOF alleles) | ISR / TLR |
| Li & al.[47] | 2019 | Asian | 150 | Not specified | TaqMan | ADIPOQ | +45T/G | Angiographic ISR |
| Li & al.[47] | 2019 | Asian | 150 | Not specified | TaqMan | ADIPOQ | +276G/T | Angiographic ISR |
