# Supplementary material for: Molecular profiling of coronary stent restenosis: A systematic review and functional analysis of implicated genes
Source: Medicine (Baltimore). 2026 Jun 26;105(26):e49455. doi: 10.1097/MD.0000000000049455 (PMC13313781; doi:10.1097/MD.0000000000049455)
Supplement: Supplementary file 6 [file medi-105-e49455-s006.docx]

**Domain-level judgments of study quality**

Item-level analysis of the Q-GENIE assessment demonstrated moderate to strong internal consistency across most domains. Higher item–total correlations were observed for domains related to statistical methods and control for confounding (0.81), technical classification of genetic variants (0.73), and appropriateness of inferences drawn (0.77). Lower correlations were noted for domains addressing other sources of bias (0.32) and testing of genetic assumptions (0.44), reflecting variability in reporting practices across studies.

These findings underscore the importance of cautious interpretation of genetic association results in ISR research and further justify the use of an evidence-weighted, descriptive synthesis approach. (Table2)

*Title: Domain-level judgments of study quality*

*Supplementary table 03: Domain-level judgments of study quality using Q genie*

| Q-GENIE Domain | Median item–total correlation | Interpretation |
| --- | --- | --- |
| Study rationale and hypothesis clarity | 0.62 | Generally adequate framing of research questions |
| Outcome definition and classification | 0.68 | Consistent definition of ISR outcomes across studies |
| Selection and comparability of groups | 0.55 | Moderate risk of selection bias |
| Technical classification of genetic variants | 0.73 | Good reporting of genotyping methods |
| Non-technical exposure classification | 0.47 | Variable handling of exposure misclassification |
| Disclosure of other sources of bias | 0.32 | Limited discussion of potential biases |
| Sample size and statistical power | 0.69 | Frequent issues with underpowered analyses |
| A priori planning of analyses | 0.51 | Moderate adherence to predefined analytical plans |
| Statistical methods and confounder control | 0.81 | Strongest methodological domain across studies |
| Testing of genetic assumptions | 0.44 | Inconsistent reporting of analytical assumptions |
| Appropriateness of inferences | 0.77 | Conclusions generally supported by results |
