# Supplementary material for: Molecular profiling of coronary stent restenosis: A systematic review and functional analysis of implicated genes
Source: Medicine (Baltimore). 2026 Jun 26;105(26):e49455. doi: 10.1097/MD.0000000000049455 (PMC13313781; doi:10.1097/MD.0000000000049455)
Supplement: Supplementary file 7 [file medi-105-e49455-s007.docx]

*Title: of genes following the main mechanistic axis.*

*Supplementary table 05: distribution of genes following the main mechanistic axis.*

| **Gene** | **Mechanistic axis** | **No. of studies** | **Total sample size*** | **Populations** | **DES-specific evidence** | **Direction of association** | **Evidence scores (0–8)** | **Evidence tier** |
| --- | --- | --- | --- | --- | --- | --- | --- | --- |
| **NOS3 (eNOS)** | Endothelial dysfunction / vascular regulation | 4 | 1,142 | White, Asian | Yes | Consistent | **7** | **High** |
| **CTGF** | Extracellular matrix remodeling / fibrosis | 1 | 657 | White | Mixed | Consistent | **6** | **High** |
| **AGT** | Renin–angiotensin system / inflammation | 1 | 175 | White | Yes | Consistent | 5 | Moderate |
| **REN** | Renin–angiotensin system / inflammation | 1 | 175 | White | Yes | Consistent | 5 | Moderate |
| **MMP3** | Extracellular matrix remodeling | 1 | 818 | Asian | Yes | Consistent | 4 | Moderate |
| **CYP2C19** | Drug metabolism / platel& response | 1 | 111 | Asian | Yes | Consistent | 4 | Moderate |
| **VEGF** | Endothelial function / angiogenesis | 1 | 120 | White | Yes | Consistent | 3 | Exploratory |
| **TGFB3** | Extracellular matrix remodeling | 1 | 172 | White (Russia) | Mixed | Not assessable | 3 | Exploratory |
| **ADIPOQ (APN)** | Metabolic / adipokine regulation | 2 | 300 | Asian | No | Inconsistent | 3 | Exploratory |
